# Electrophysiological responses to negative evaluative person-knowledge: Effects of individual differences

**DOI:** 10.3758/s13415-021-00894-w

**Published:** 2021-04-13

**Authors:** Claudia Krasowski, Sebastian Schindler, Maximilian Bruchmann, Robert Moeck, Thomas Straube

**Affiliations:** 1 Institute of Medical Psychology and Systems Neuroscience, University of Muenster, Von-Esmarch-Str. 52, D-48149 Münster, Germany; 2 Otto Creutzfeldt Center for Cognitive and Behavioral Neuroscience, University of Muenster, Münster, Germany

**Keywords:** Evaluative knowledge, Feature-based attention, EEG/ERP, Individual threat-sensitivity, Trait anxiety, Neuroticism, Agreeableness

## Abstract

Faces transmit rich information about a unique personal identity. Recent studies examined how negative evaluative information affects event-related potentials (ERPs), the relevance of individual differences, such as trait anxiety, neuroticism, or agreeableness, for these effects is unclear. In this preregistered study, participants (*N* = 80) were presented with neutral faces, either associated with highly negative or neutral biographical information. Faces were shown under three different task conditions that varied the attentional focus on face-unrelated features, perceptual face information, or emotional information. Results showed a task-independent increase of the N170 component for faces associated with negative information, while interactions occurred for the Early Posterior Negativity (EPN) and the Late Positive Potential (LPP), showing ERP differences only when paying attention to the evaluative information. Trait anxiety and neuroticism did not influence ERP differences. Low agreeableness increased EPN differences during perceptual distraction. Thus, we observed that low agreeableness leads to early increased processing of potentially hostile faces, although participants were required to attend to a face-unrelated feature.

**Supplementary Information:**

The online version contains supplementary material available at 10.3758/s13415-021-00894-w.

## Introduction

Faces are a significant part of communication, which transmit rich and unique identity-information about a person. Such knowledge can be threatening, for example knowing that it is a face of a criminal who committed a horrible crime. Such negative information leads not only to differential face processing, but it has been suggested that there are individual differences in the acquisition and maintenance of threatening associations (Lonsdorf & Merz, 2017), as well as individual differences in the processing of such negative, in particular, threat-related information (for trait anxiety, e.g., Bar-Haim et al., 2005; for neuroticism, e.g., Doty et al., 2013; for agreeableness, e.g., Meier et al., 2006). Trait anxiety and neuroticism are highly correlated (Bishop & Forster, 2013), and both have been linked to an increased sensitivity to detect faces signaling threat (Chan et al., 2007; Doty et al., 2013; Andric et al., 2016; de Jong et al., 2009). Furthermore, neuroticism and agreeableness have been found to intensify behavioral evaluative effects (Vogel et al., 2019), showing more negative ratings of faces that were associated with negative picture information. Agreeableness also has been found to influence the evaluation of facial expressions (Czerwon et al., 2011; Knyazev et al., 2008). Violent offenders, exhibiting very low agreeableness scores, show a bias to detect anger in angry-fearful ambiguous expressions (Wegrzyn et al., 2017). Thus, low agreeable individuals are sensitive to detect hostility in faces, whereas individuals with high anxiety and neuroticism scores are found to be sensitive to detect signs of danger. However, while individual differences in threat-related information processing are hypothesized (Bar-Haim et al., 2005), we are not aware of any study testing such differences in processing threatening information based on evaluative knowledge, and it is yet unknown whether these traits are related to biased processing of threatening faces where threat information is derived by evaluative background stories.

Event-related potentials (ERP) can be used to examine biased face processing, because ERPs index different early and late stages of face and emotion processing. The occipitally scored P1 reflects an early stage of stimulus detection and discrimination (Hopfinger & Mangun, 1998; Luck & Hillyard, 1994; Vogel & Luck, 2000). The following N170 amplitude is viewed as a structural encoding component and the amplitude increases for faces compared with objects and is further magnified by threatening compared to neutral expressions (Eimer, 2011; Hinojosa et al., 2015). The subsequent Early Posterior Negativity (EPN) is observed as differential negativity when contrasting emotional and neutral expressions and has been related to early attentional selection (Schupp et al., 2004; Wieser et al., 2010). Finally, the Late Positive Potential (LPP) arises as differential positivity for emotional information and indicates stimulus evaluation and controlled attention processes (Hajcak et al., 2009; Harald Thomas Schupp et al., 2006).

Recent studies show that contextual information influence ERPs, even for neutral faces (Bublatzky et al., 2017; Bublatzky et al., 2020a, b; for a review, see Wieser & Brosch, 2012). However, findings are inconsistent, which might be due to differences in experimental tasks and examined ERPs. Studies that examine evaluative information effects for neutral expressions rarely examined early ERP components. The only one study including the P1 component so far showed no modulations (Luo et al., 2016), while two studies are reporting conflicting findings regarding the N170 (Luo et al., 2016; Xu et al., 2016). For the EPN, amplitudes are frequently observed to be more negative-going for faces paired with negative compared with neutral biographical information (Abdel Rahman, 2011; Luo et al., 2016; Suess et al., 2015; Xu et al., 2016), whereas some studies or conditions showed no EPN effects (Kissler & Strehlow, 2017; Baum et al., 2018; for unfamiliar faces Abdel Rahman, 2011). For the LPP, the majority of studies showed larger amplitudes for negatively associated faces (Abdel Rahman, 2011; Kissler & Strehlow, 2017; Baum et al., 2018; Xu et al., 2016; but see Luo et al., 2016). To summarize, studies showed that evaluative information modulates ERPs, but provided a mixed picture of which information processing stages are affected. We recently suggested that task conditions might affect ERPs in studies manipulating evaluative person knowledge when stimulus viewing time is limited (Schindler et al., in press). In particular, we found that task-independent effects of negative evaluative information for the N170, while EPN and LPP were potentiated when the evaluative information became task-relevant (Schindler et al., in press). How individual traits affect ERPs to faces associated with negative person knowledge in general and how effects are modulated by task conditions, in particular, are unknown.

Several studies have investigated the relationship between trait anxiety and responses to fearful or angry face information and reported increased early ERP components for participants with high trait anxiety (for the P1, see Bar-Haim et al., 2005; Holmes et al., 2008; for the N170, see Williams et al., 2007; but see Morel et al., 2014; Walentowska & Wronka, 2012). Some studies show rather reduced EPN (Holmes et al., 2008; Walentowska & Wronka, 2012) or no influences of trait anxiety on P1, N170, or EPN amplitudes (Morel et al., 2014). This variability might be rooted in the used attention tasks, because EEG and fMRI findings suggest that individual sensitivity to threatening information might be most pronounced in implicit emotion processing conditions (Straube et al., 2004; Quarto et al., 2014; for a review see Straube et al., 2011). For example, studies reported increased N170 (Williams et al., 2007) or decreased EPN amplitudes among high trait anxious subjects for masked fearful facial expressions (Walentowska & Wronka, 2012), while another study failed to find a trait anxiety-related increase of any early ERP amplitudes for fearful faces in an emotion discrimination task (Morel et al., 2014). For later components, attenuated processing of fearful faces for high trait anxiety is more frequently reported, while here attention tasks varied from attention to the face, the emotion or masked face presentation (Holmes et al., 2008; Morel et al., 2014; Walentowska & Wronka, 2012). To summarize, some studies point to an anxiety-related early increase of ERPs towards threatening faces during implicit attention tasks, which might suggest an initial hypersensitivity to threat irrespective of attention. The later reduced differential processing might be either due to avoidance of threatening stimuli or due to overgeneralization and thus a strong responding to all stimuli, including neutral stimuli (for example, see Onat & Büchel, 2015). For neuroticism and agreeableness, studies examining ERPs towards threatening faces are missing. While no studies are examining evaluative face processing in neuroticism or agreeableness, studies using words or pictures showed increased processing of negative content at late processing stages for high neurotic participants (Gomez et al., 2002; Zhang et al., 2013; Ku et al., 2020; Zhang et al., 2015; but see Bartussek et al., 1996; Speed et al., 2015). Participants exhibiting a high degree of callous-unemotional (CU) traits, which is related to low agreeableness, show reduced N170 or LPP responses to fearful expressions (Brislin et al., 2018; Brislin & Patrick, 2019).

In summary, evaluative person knowledge modulates ERPs, while conflicting findings might be related to the attention task (e.g., reporting LPP effects Baum et al., 2018; reporting no effects, see Luo et al., 2016). Furthermore, while trait anxiety, neuroticism, or agreeableness might further add to negative, in particular, threat-related information processing (for trait anxiety, e.g., Bar-Haim et al., 2005; for neuroticism, e.g., Doty et al., 2013; for agreeableness, e.g., Meier et al., 2006), there are yet no studies which examined individual differences on the processing of faces associated with negative evaluative person knowledge. In this preregistered study (https://osf.io/pt5sd), we examined how early (P1, N170), mid-latency (EPN), or late (LPP) ERPs are affected by evaluative knowledge under different attentional tasks. Participants had to respond to faces associated with highly aversive or neutral biographical information in three different tasks: i) a perceptual line discrimination task, ii) an age decision task, where attention was directed to the face, and iii) an emotional decision task, where attention was directed to the evaluative face information. We expected no P1 effects of evaluative emotional information, but for the N170 and EPN, we expected increased processing of negative faces when attention was directed to the face and emotional information. We expected differential LPP effects only in the emotion task. Importantly, our main study goal was to explore possible influences of trait anxiety, neuroticism, and agreeableness on ERP differences in a large sample (*N* = 80). We had no a priori predictions regarding individual differences in ERP responses but provide an overview of if relationships exist and, if so, how they depend on the attention task.

## Methods

### Participants

In total, 85 participants were recruited at the University of Münster. One participant had to be excluded due to a neurological disorder, two due to bad EEG data recording, and two due to incomplete behavioral data. The remaining registered 80 participants (58 females) were 23.90 years on average (range 18–34 years, standard deviation [*SD*] = 3.26) and fulfilled the registered data sampling plan.^1^ For such a sample size, power calculations using G*Power 3.1.7 (Faul et al., 2009) showed a high power (>99%) to detect medium effect sizes, which were expected for correlations of individual differences and ERP differences (around *r* = 0.25 for between method correlations, see Hemphill 2003). All participants gave written informed consent and received 10 Euros per hour for participation. All had normal or corrected-to-normal vision, were right-handed, and had no self-reported history of neurological or psychiatric disorders. Please note that a previous study using a smaller initial subsample of the current sample was dedicated to the task-based ERP modulations only (Schindler et al., in press).

### Stimuli and questionnaires

The facial stimuli for the experiment were taken from the FACES database with permission for use in the current experiment (Ebner et al., 2010). Faces of eight identities (4 young males, 4 middle-aged males) showed neutral facial expressions. Half of the faces were paired with highly negative evaluative knowledge and the other half with neutral information (see below).^2^ Identities were counterbalanced assigned to the conditions and the composition of groups was balanced across participants. To ensure face-validity when coupling the faces with different pieces of narrative information, we displayed only head-shots of each face, keeping the facial hair but removing the shirts. Faces were always displayed with an overlay of horizontal or vertical thin lines. Lines were overlaid to the faces using presentation (www.neurobehavioralsystems.org), showing five horizontal or vertical lines within the boundaries of the face (horizontal lines 1.4 lengths; vertical lines 2.0 length; thickness 0.01; centered around x = 0, y = 0).

Participants completed the Becks Depression Inventory-II (BDI-II, Hautzinger et al., 2009), the Spielberger State-Trait Anxiety Inventory (STAI; Spielberger et al., 1999), and a short version of the NEO Five-Factor Inventory (NEO-FFI, Körner et al., 2008). Descriptive values and intercorrelations between questionnaires are reported in Table 1. Unsurprisingly, there are strong relationships between measures of depression, anxiety, and personality, most pronounced for the relationship between neuroticism and trait anxiety (Table 1). The items of the 30-item NEO-FFI short version are scaled as a Likert-type scale, with five possible answers (*strongly disagree* – *strongly agree*), and in our sample mean and Cronbach’s alpha scores are similar to those reported in the German normative study (NEO-FFI, Körner et al., 2008).

**Table 1:** All examined questionnaires and intercorrelations

Questionnaire	Mean (SD)		Min / Max		Cronbach´s α		25 / 50 / 75 Quartile	
BDI-II	6.04 (5.21)		0 / 23		0.847		2 / 5 / 9	
STAI state	34.80 (7.04)		21 / 57		0.869		30 / 34 / 38.75	
STAI trait	37.42 (7.74)		26 / 66		0.893		32 / 36 / 41.75	
neuroticism	1.27 (0.75)		0.17 / 3.83		0.794		0.71 / 1.25 / 1.67	
extraversion	2.55 (0.60)		0.67 / 3.67		0.747		2.17 / 2.67 / 3.00	
openness	2.53 (0.78)		0.17 / 3.83		0.764		2.00 / 2.58 / 3.13	
agreeableness	3.04 (0.58)		1.33 / 4.00		0.677		2.67 / 3.08 / 3.50	
conscientiousness	3.10 (0.63)		1.17 / 4.00		0.809		2.83 / 3.33 / 3.50	
Pearson correlations	(1)	(2)	(3)	(4)	(5)	(6)	(7)	(8)
BDI-II (1)	1							
STAI state (2)	0.340	1						
STAI trait (3)	0.582*	0.442	1					
neuroticism (4)	0.557	0.353	0.829	1				
extraversion (5)	−0.093	−0.044	-0.197	−0.0132	1			
openness (6)	−0.004	0.162	0.183	0.041	0.155	1		
agreeableness (7)	−0.395	−0.257	−0.327	−0.238	0.290	−0.015	1	
conscientiousness (8)	−0.512	−0.273	−0.363	−0.275	0.153	−0.213	0.336	1

^a^ Bonferroni-adjusted significance threshold for 28 intercorrelations is *p* < 0.001786.

*p < 0.0017.

### Procedure

**Fig. 1:**
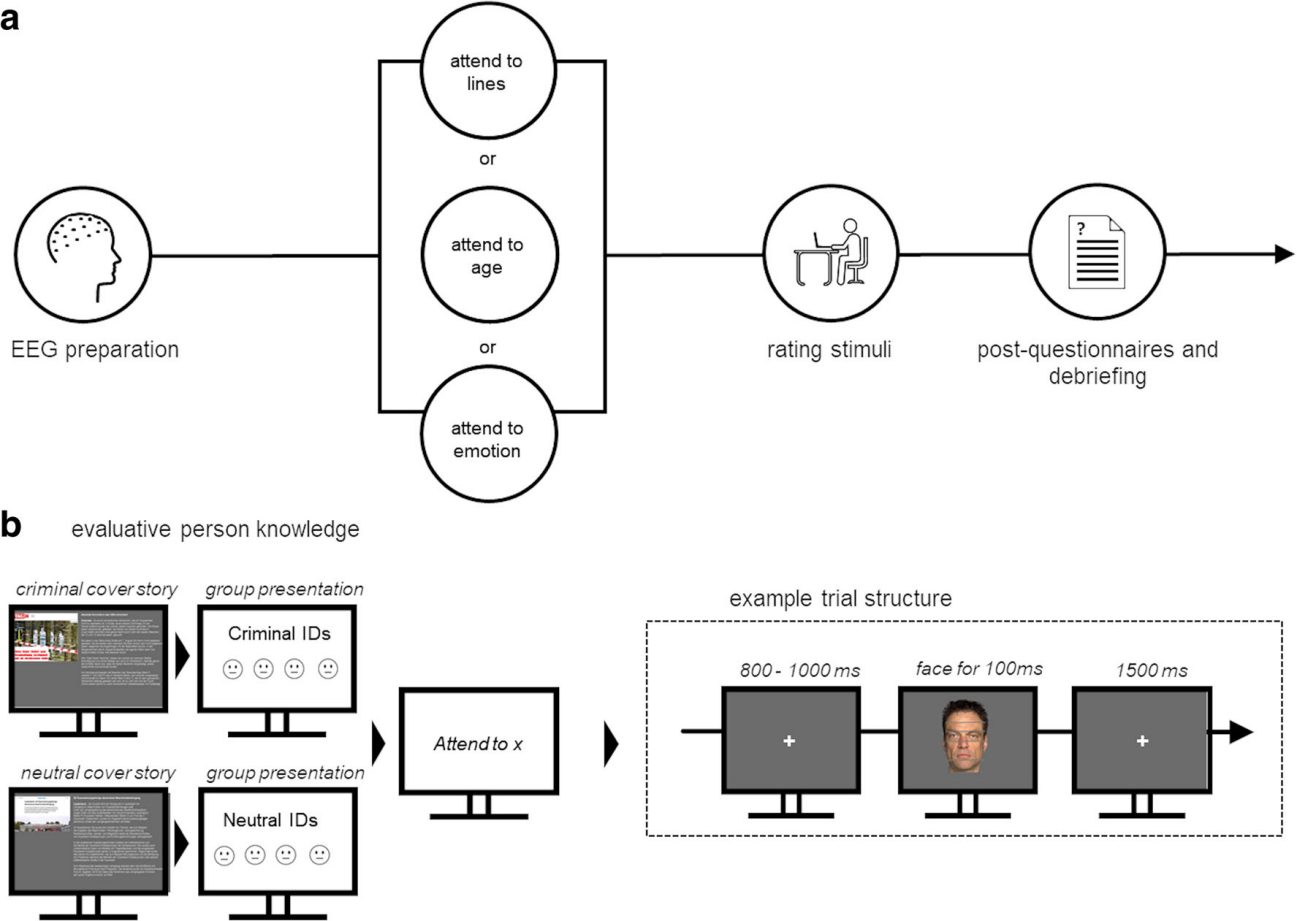
Experiment overview. **a)** Schematic overview of the experimental flow. **b)** Before each attention task, first the background story on the criminal group and then the story on the firefighter group was presented, after which the four faces for each group were displayed

Participants were seated 60 cm in front of a Gamma-corrected display (Iiyama G-Master GB2488HSU) running at 60 Hz with a Michelson contrast of 0.9979 (*L*_*min*_ = 0.35 cd/m^2^; *L*_*max*_ = 327.43 cd/m^2^). The background was set to medium grey (RGB 108, 108, 108). Participants were given the negative and neutral background information of the eight identities, showing newspaper headlines and a detailed explanation. Two young and middle-aged males were portrayed to have committed a brutal crime—raping, mutilating, and killing two young females. The other two young and middle-aged males were portrayed to have participated in firefighter training. After each newspaper article, all individual faces were presented at the screen, requesting participants to attentively look at all faces and memorize them (see Fig. 1b, for the translated background stories see the Supplementary Material). Consecutively, participants started with the perceptual, age, or evaluative decision task (Fig. 1). Participants were instructed to avoid eye-movements and blinks during the stimulus presentation. Task order and response buttons (x and m) were counterbalanced. In each trial, participants always had to decide in a two-alternative forced-choice task: i) if overlaid line orientation was horizontal or vertical, or ii) if the age of the face was old or young, or iii) if the evaluative information was negative or neutral, i.e., if the face belongs to the criminal or firefighter-training group. Before each task started, the group information was repeated to ensure learning of the evaluative information and the corresponding faces. The trial structure was constant across all tasks. Each trial started with the display of a fixation cross for 800 to 1,000 ms, after which a face was presented for 100 ms. The display of the face was followed by another fixation cross, which was presented for 1,500 ms, during which the responses were recorded. Each face was repeated during each task 16 times, leading in total to 64 trials presenting faces with evaluative negative information and 64 trials presenting faces with evaluative neutral information, summing up to a total of 384 trials. After the main experiment, participants rated each face in valence, arousal, and perceived threat and responded to a demographic questionnaire, the BDI-II, the STAI, and the NEO-FFI (Hautzinger et al., 2009; Körner et al., 2008; Spielberger et al., 1999).

Then, instructions for the respective attention task were given. An example trial for all three tasks is provided. Please note that proportions were increased to increase visibility. The depicted face was not used in the experiment but is available for public display.

### EEG recording and preprocessing

EEG signals were recorded from 64 BioSemi active electrodes using Biosemis Actiview software (www.biosemi.com). Four additional electrodes measured horizontal and vertical eye-movements. The recording sampling rate was 512 Hz. Offline data were re-referenced to average reference and filtered with a low-cutoff filter of 0.01 (6 dB/oct) and a 40 Hz low-pass zero-phase filter (24 dB/oct). Recorded eye movements were corrected using the automatic eye-artifact correction method implemented in BESA (Ille et al., 2002). A predefined source model was applied to the data, combining three topographies accounting for EOG activities, consisting of horizontal and vertical eye-movement and blinks (HEOG, VEOG, blink) with 12 regional sources modeling the different brain regions. The adaptive artifact correction method then performed a principal component analysis (PCA) for segments where the correlation between data and artifact topography exceeded the HEOG (150 μV) or VEOG (250 μV) thresholds. All PCA components explaining more than the minimum variance were maintained and then recorded data decomposed using all topographies into a linear combination of brain and artifact activities (Ille et al., 2002). The remaining artifacts were rejected based on an absolute threshold (<120 μV), signal gradient (<75 μV/∂T), and low signal (i.e., the *SD* of the gradient, >0.01 μV/∂T). Noisy EEG sensors were interpolated using a spline interpolation procedure. A delay of the LCD screen for stimulus presentation of 15 ms, measured by a photodiode, was corrected during epoching. Filtered data were segmented from 100 ms before stimulus onset until 1,000 ms after stimulus presentation. Baseline-correction used the 100 ms before stimulus onset. On average, 5.23 electrodes were interpolated and 19 percent of trials were rejected. On average this resulted in 51.90 neutral and 51.79 negative face trials during the perceptual, 51.24 neutral and 51.29 negative face trials during the age, and 51.36 neutral and 52.73 negative face trials during the perceptual emotion task. For the number of kept trials, no differences regarding emotion (*F*_(1,79)_ = 2.05, *p* = 0.156, η_P_^2^ = 0.025) or task were found (*F*_(1.78,140.49)_ = 0.39, *p* = 0.654, η_P_^2^ = 0.005) and there was no interaction (*F*_(2,158)_ = 2.22, *p* = 0.113, η_P_^2^ = 0.027).

### Data analyses

Our main study goal was to test the relationship of ERP components to three different individual trait scores (trait anxiety, neuroticism, and agreeableness scores). To this end, trait scores were correlated with the obtained differences between negative and neutral faces using JASP (www.jasp.org). We calculated both Bonferroni-corrected inferential (adjusted *p*-value for 36 correlations <0.001388) and Bayesian Pearson correlation coefficients. For Bayesian analyses, the null hypothesis was specified as a point-null prior (i.e., standardized effect size *δ* = 0) and defined the alternative hypothesis as a Jeffreys-Zellner-Siow (*JZS*) prior, i.e., a folded Cauchy distribution centered around *δ* = 0 with the scaling factor *r* = 0.707. This scaling factor assumes a roughly normal distribution. To assign verbal labels to the strength of evidence, we followed the taxonomy suggested by Jeffreys (1998), labeling Bayes Factors with a BF_10_ of 1 as no evidence, BF_10_ between 1-3 as anecdotal evidence, 3-10 as moderate evidence, 10-30 as strong evidence, 30-100 as very strong evidence, and larger BFs as extreme evidence in favor of the alternative hypothesis (H1) over the null hypothesis (H0). The reverse BF_01_ labels evidence for the null hypotheses (BF_10_ = 1/ BF_01_). Finally, for exploratory purposes, unregistered linear regressions including all questionnaires are reported in the Supplement.

Secondary analyses were carried out to validate expected behavioral and EEG scalp data effects. For rating data, paired *t*-tests were calculated between faces associated with negative and neutral information. For all other analyses, two (evaluative information: negative, neutral) by three (task: perceptual, age, evaluative emotional information) repeated measure ANOVAs were calculated. Partial eta-squared (partial η^2^) was estimated to describe effect sizes (Cohen, 1988). When Mauchly’s test indicated a violation of sphericity, degrees of freedom were corrected according to Greenhouse-Geisser. Reaction times below 100 ms and above 1500 ms were not regarded as correct responses (‘hit’). Behavioral responses were recoded when accuracy was close to ceiling but codes were reversed (three times in the evaluative task, seven times in the age task). Incomplete rating and behavioral data from two participants led to their above-described exclusion (see participants section).

Time windows were segmented into intervals from 80 to 100 ms for the P1, from 120 to 170 ms for the N170, from 250 to 350 ms for the EPN, and from 400 to 600 ms for the LPP. We measured the P1, N170, and EPN over two symmetrical occipital clusters (P1 and N170:

P9, P7, PO7, P10, P8, PO8; EPN: P9, P7, PO7, O1, P10, P8, PO8, O2). Additionally, we measured the LPP component over a centro-parietal cluster (C1, Cz, C2, CP1, CPz, CP2, P1, Pz, P2). We registered to validate ERP windows for the P1 and N170 by collapsing ERPs across all conditions (Luck & Gaspelin, 2017). For the EPN and LPP, typically scored as differences between emotional and neutral stimuli, we collapsed negative faces and neutral faces across all attention tasks to identify differential effects. This led us to slightly deviate from our preregistration in time (registered N170: 130 to 170 ms; EPN 200 to 350 ms) and space (registered EPN: left P9, P7, PO7; right P10, P8, PO8; LPP: C1, Cz, C2, CP1, CPz, CP2).

## Results

### Behavioral results and rating data

**Fig. 2:**
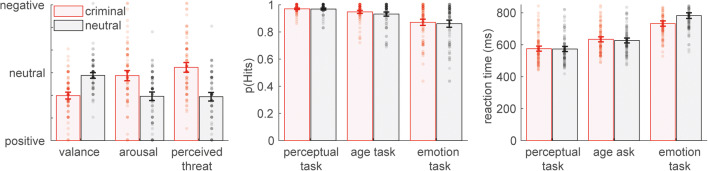
Ratings and behavioral results across the three attention tasks. Error bars show 95% confidence intervals

Participants rated all faces according to valence, arousal, and perceived threat after the experiment but before debriefing. The face to be evaluated was presented and rated on a scale from 1 to 7 (1 = low, 4 = neutral, 7 = high positive valence, high arousal, or high perceived threat). For valence, faces paired with neutral information had been rated significantly higher than faces paired with negative information (*t*_(79)_ = 6.39, *p* < 0.001; Fig. 2). Both arousal (*t*_(79)_ = 5.32, *p* < 0.001) and perceived threat (*t*_(79)_ = 6.80, *p* < 0.001) were judged higher for faces associated with negative information compared with neutral faces.

Overall hit rate was 93%, showing no main effect of evaluative information (*F*_(1,79)_ = 3.20, *p* = 0.077, partial η^2^ = 0.039; Fig. 2; Table 2) but a main effect of task (*F*_(1.37,107.91)_ = 41.85, *p* < 0.001, partial η^2^ = 0.346). Hit rate concerning the evaluative decision task was lower compared with the age decision task (*p* < 0.001) and the perceptual task (*p* < 0.001). Furthermore, hit rate was higher in the perceptual task compared to the age decision task (*p* < 0.001; Table 2). There was no significant interaction between evaluative information and task (*F*_(1.78,140.40)_ = 0.63, *p* = 0.517, partial η^2^ = 0.008). Regarding reaction time, main effects of evaluative information (*F*_(1,79)_ = 19.03, *p* < 0.001, partial η^2^ = 0.194) and of task were found (*F*_(1.77,139.47)_ = 257.76, *p* < 0.001, partial η^2^ = 0.765). Reaction times were significantly shorter in the perceptual task compared to both the age decision task (*p* < 0.001) and the evaluative decision task (*p* < 0.001). In the age task, participants demonstrated also significant faster reaction times than in the evaluative task (*p* < 0.001; Table 2). There was a significant interaction between evaluative information and task (*F*_(1.62,127.68)_ = 33.19, *p* < 0.001, partial η^2^ = 0.296). Post-hoc tests showed a larger difference between negatively and neutrally associated faces for the evaluative compared to the perceptual task (*p <* 0.001) and for the evaluative compared to the age task (*p <* 0.001) but not between the age and the perceptual task (*p =* 0.361; Table 2; Fig. 2).

**Table 2:** Behavioral results across the three attention tasks

	Perceptual task		Age task		Evaluative task	
	Neutral faces	Criminal faces	Neutral faces	Criminal faces	Neutral faces	Criminal faces
Accuracy in hits (*SD*)	0.97 *(0.04)*	0.97 *(0.03)*	0.93 *(0.08)*	0.95 *(0.06)*	0.86 *(0.14)*	0.87 *(0.13)*
Reaction time in ms (*SD*)	573 *(88)*	575 *(88)*	627 *(84)*	634 *(86)*	782 *(96)*	*732 (90)*

Hits are displayed in percent correct. Reaction times are rounded to milliseconds.

### ERP results

#### **P1**

**Fig. 3:**
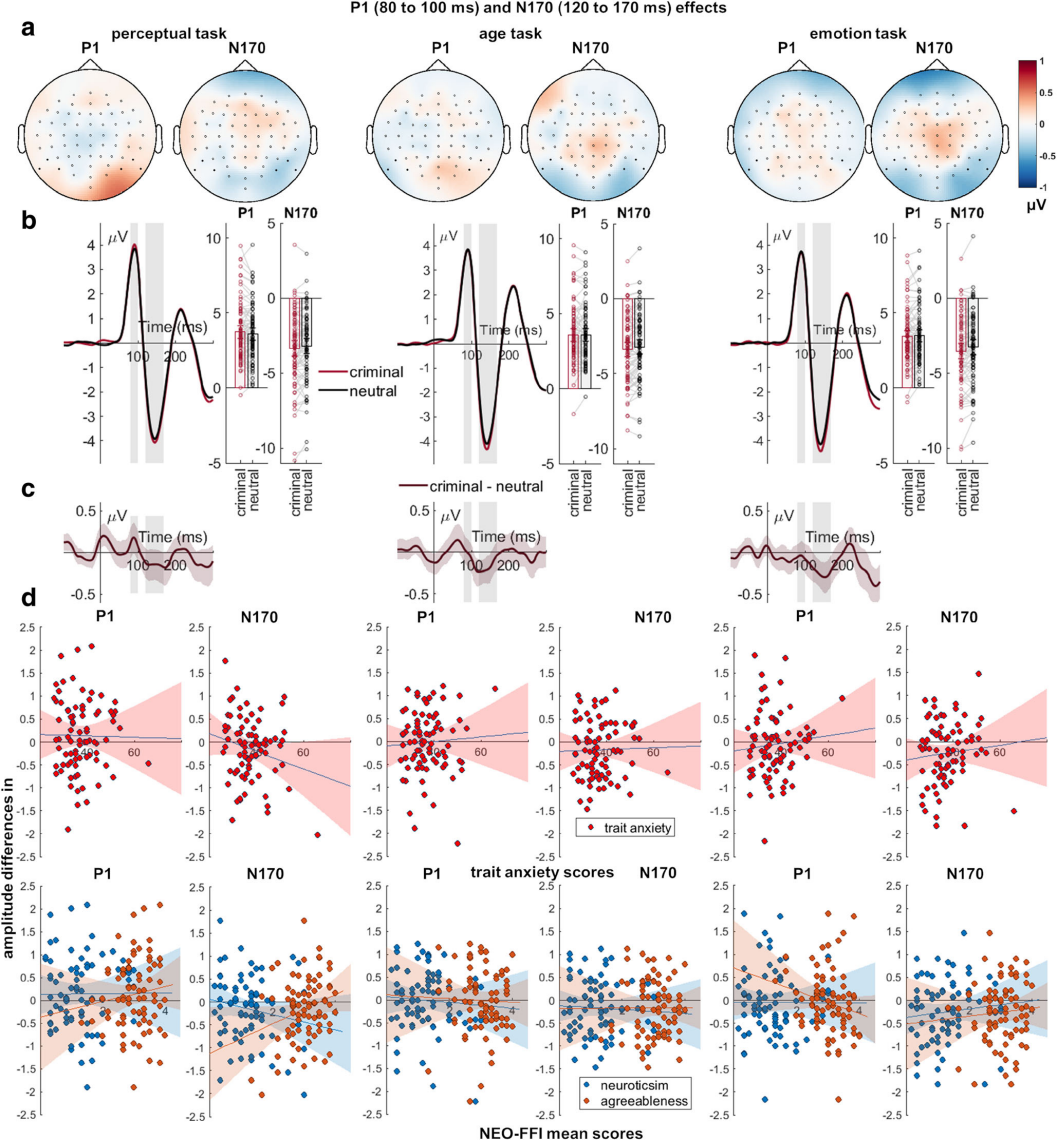
Effects of evaluative information for the P1 and N170. **A)** Scalp topographies depict the differences between criminal and neutral faces. **B)** ERP waveforms show the time course for highlighted sensors. Bar charts show mean and individual data points, and error bars show 95% confidence intervals. **C)** Respective difference plots contain 95% bootstrap confidence intervals of intra-individual differences. **D)** Scatter plots of P1 and N170 differences with anxiety, neuroticism, and agreeableness scores. The 95% confidence intervals are highlighted

There was no effect of evaluative information, but a significant effect of task (Table 3; Fig. 3). For the main effect of task, smaller P1 amplitudes were recorded in the evaluative information task compared to the perceptual task (*p* = 0.001) and age task (*p* = 0.044). There was no interaction between evaluative information and task. While Bayes Factors showed inconclusive evidence for a relationship between agreeableness and P1 amplitude differences in the emotion task (Table 4), whereas all further correlation analyses showed moderate evidence for the absence of a relationship between trait anxiety, neuroticism, or agreeableness and differential P1 effects (BF_01_ > 4).

**Table 3:** Results from 2 × 3 repeated measures ANOVAs for each ERP-component

	Effect	DF, DFe	ANOVA results		
			*F*	*P*	η_P_^2^
P1	Evaluative	1, 79	0.07	0.792	0.001
	task	2, 158	**6.63**	**0.002**	**0.077**
	Evaluative x task	2, 158	1.77	0.174	0.022
N170	Evaluative	1, 79	**17.63**	**<0.001**	**0.182**
	task^a^	2, 158	0.99	0.370	0.012
	Evaluative x task	2, 158	0.80	0.448	0.010
EPN	Evaluative	1, 79	**5.30**	**0.024**	**0.063**
	task^a^	2, 158	**10.60**	**<0.001**	**0.118**
	Evaluative x task	2, 158	**3.10**	**0.048**	**0.038**
LPP	Evaluative	1, 79	**26.22**	**<0.001**	**0.249**
	task^a^	2, 158	**12.48**	**<0.001**	**0.136**
	Evaluative x task^a^	2, 158	**8.19**	**0.001**	**0.094**

Effects with the letter ^a^*, p*-values were Greenhouse-Geisser-corrected, because Mauchly tests indicated a violation of the sphericity assumption. Significant main and interaction effects are highlighted in bold font. Evaluative refers to effects of evaluative (negative/neutral) biographical information.

**Table 4:** P1 and N170 correlations with individual trait scores

P1	Correlation	Perceptual task	Age task	Emotion task
Trait anxiety	Pearson's *r*	0.011	0.087	0.109
	*p*-value^a^	0.920	0.444	0.337
	BF_10_	0.14	0.19	0.22
Neuroticism	Pearson's *r*	0.034	−0.038	0.017
	*p*-valuea	0.766	0.739	0.883
	BF_10_	0.15	0.15	0.14
Agreeableness	Pearson's *r*	0.120	-0.038	−0.225
	*p*-value^a^	0.291	.738	.045
	BF_10_	0.24	0.15	1.00
N170	Correlation	Perceptual task	Age task	Emotion task
Trait anxiety	Pearson's *r*	−0.197	0.036	0.096
	*p*-value^a^	0.080	0.752	0.399
	BF_10_	0.63	0.15	0.20
Neuroticism	Pearson's *r*	−0.153	−0.058	0.105
	*p*-value^a^	0.174	0.608	0.355
	BF_10_	0.35	0.16	0.21
Agreeableness	Pearson's *r*	0.279	0.005	0.068
	*p*-value^a^	0.012	0.967	0.548
	BF_10_	3.09	0.14	0.17

^a^ Bonferroni-corrected significance threshold is *p* < 0.001388. BF_10_ indicates evidence in favor of the alternative hypothesis (H1) and conversely BF_01_ evidence in favor of the null hypothesis (where BF_10_ = 1/ BF_01_).

#### **N170**

For the N170, a large main effect of evaluative information but no main effect of task was found (Table 3; Fig. 3). Faces paired with negative information elicited larger N170 amplitudes than those with neutral information. There was no interaction between evaluative information and task. Correlation analyses showed moderate evidence for a positive relationship between agreeableness and the N170 amplitude differences during the perceptual task (Table 4), while this failed the Bonferroni-corrected threshold. Most correlations showed moderate evidence for the absence of a relationship, while only anecdotal evidence against a relationship between trait anxiety and neuroticism during the perceptual task could be observed (BF_10_ = 0.63 and 0.35; BF_01_ = 1.59 and 2.86; Table 4). Furthermore, while an N170 relationship with agreeableness for the perceptual task failed the Bonferroni corrected significance threshold, Bayes Factors show moderate evidence for such a correlation.

#### **EPN**

**Fig. 4:**
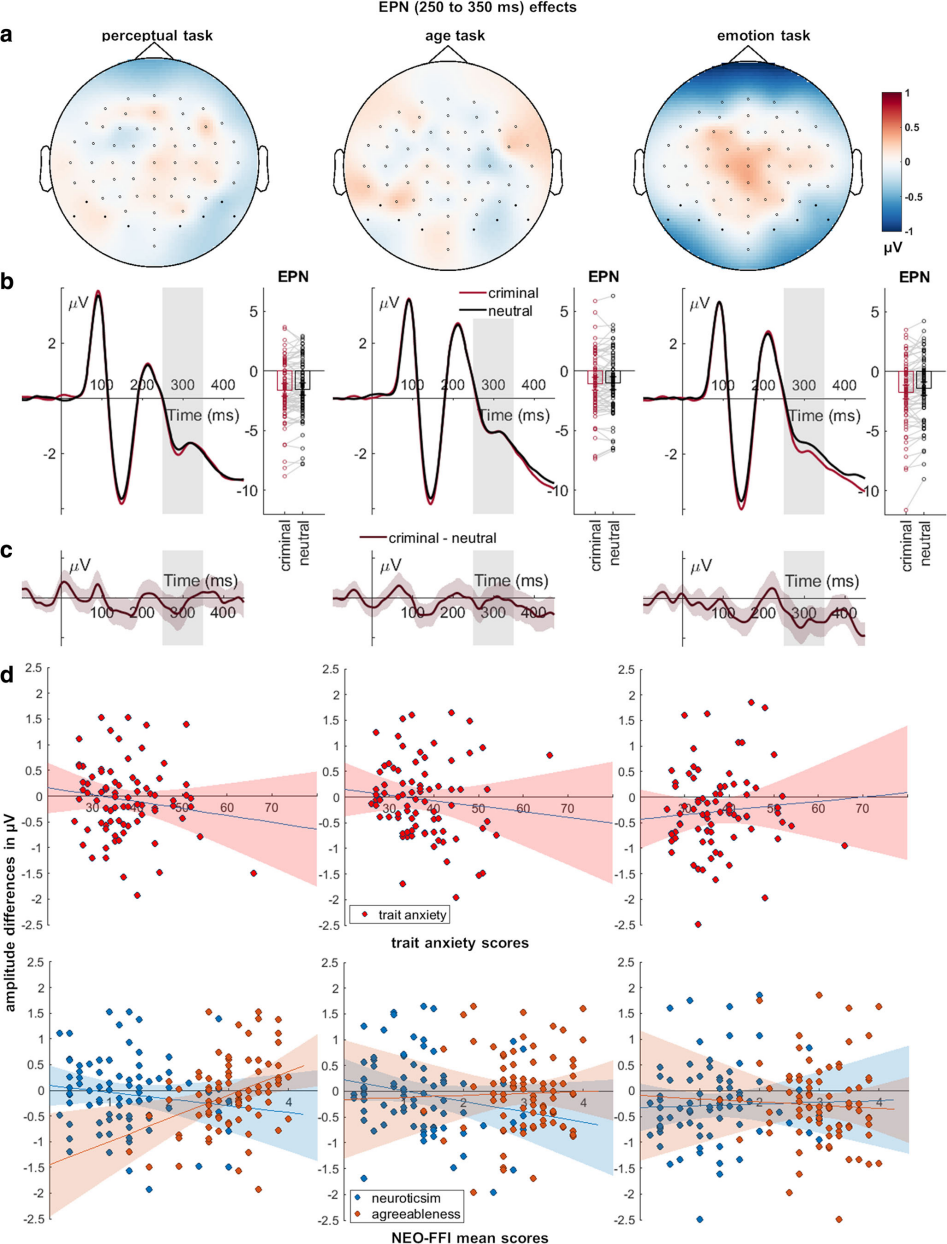
Interaction effects between evaluative information and task for the EPN. Scalp topographies depict the differences between criminal and neutral faces. **B)** ERP waveforms show the time course for highlighted sensors. **C)** Respective difference plots contain 95% bootstrap confidence intervals of intra-individual differences. **D)** Scatter plots of EPN differences with anxiety, neuroticism, and agreeableness scores. The 95% confidence intervals are highlighted

Regarding the EPN, there was a main effect of evaluative information and an effect of task (Table 3; Fig. 4). Faces paired with negative information showed larger EPN amplitudes and larger EPN amplitudes were recorded during the perceptual and evaluative task compared to the age task (*ps* ≤ 0.001). Furthermore, we observed a significant interaction between evaluative information and task. Post-hoc tests showed a larger difference between negatively and neutrally associated faces for the evaluative compared to the perceptual task (*M*_difference_ = −0.25, *SD* = 1.09; *t*_(79)_ = −2.05, *p =* 0.044). No differences were observed for the evaluative compared with the age task (*M*_difference_ = −0.27, *SD* = 1.97; *t*_(79)_ = −1.23, *p =* 0.223), or between the age and the perceptual task (*M*_difference_ = −0.02, *SD* = 1.05; *t*_(79)_ = −0.17, *p =* 0.865). The correlation analyses revealed a positive relationship between agreeableness and EPN amplitude differences in the perceptual task (Table 5; Fig. 4). For the anxiety and neuroticism scores, no such correlations were observed (Table 5; Fig. 4).

**Table 5:** EPN and LPP correlations with individual trait scores

EPN	Correlation	Perceptual task	Age task	Emotion task
Trait anxiety	Pearson's *r*	−0.117	−0.067	0.070
	*p*-value^a^	0.302	0.553	0.538
	BF_10_	0.24	0.17	0.17
Neuroticism	Pearson's *r*	−0.120	−0.160	0.043
	*p*-value^a^	0.291	0.157	0.707
	BF_10_	0.24	0.37	0.15
Agreeableness	Pearson's *r*	0.369*	<0.001	−0.041
	*p*-value^a^	**0.001**	0.998	0.717
	BF_10_	**36.51**	0.140	0.15
LPP	Correlation	Perceptual task	Age task	Emotion task
Trait anxiety	Pearson's *r*	−0.044	−0.026	−0.065
	*p*-value^a^	0.698	0.822	0.569
	BF_10_	0.15	0.14	0.16
Neuroticism	Pearson's *r*	0.050	−0.036	0.085
	*p*-value^a^	0.658	0.751	0.455
	BF_10_	0.15	0.15	0.18
Agreeableness	Pearson's *r*	0.014	0.044	−0.037
	*p*-value^a^	0.904	0.700	0.743
	BF_10_	0.14	0.15	0.15

^a^ Bonferroni-corrected significance threshold is *p* < 0.001388; *p < 0.0013 and BF_10_ > 10. BF_10_ indicates evidence in favor of the alternative hypothesis (H1) and conversely BF_01_ evidence in favour of the null hypothesis (where BF_10_ = 1/ BF_01_).

#### LPP

**Fig. 5:**
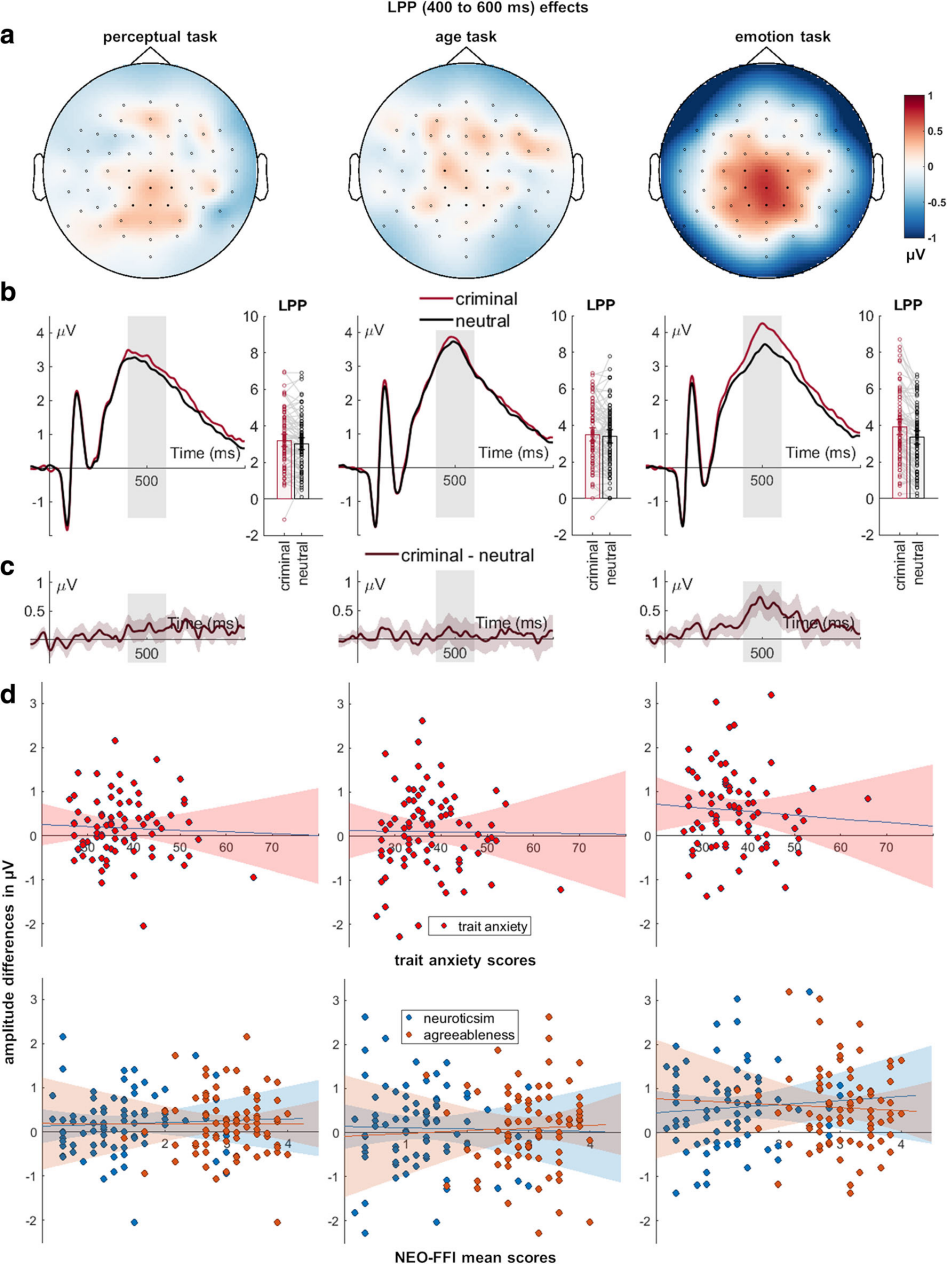
Interaction effects between evaluative information and task for the LPP. Scalp topographies depict the differences between criminal and neutral faces. **B)** ERP waveforms show the time course for highlighted sensors. **C)** Respective difference plots contain 95% bootstrap confidence intervals of intra-individual differences. **D)** Scatter plots of LPP differences with anxiety, neuroticism, and agreeableness scores. The 95% confidence intervals are highlighted

For the LPP, main effects of evaluative information and task were found (Table 3, Fig. 5). Faces associated with negative information elicited larger positivities than faces paired with neutral information. Furthermore, larger LPP amplitudes were recorded for faces presented in the evaluative task compared to the perceptual (*p* < 0.001) and in the age task compared with the perceptual task (*p* = 0.001). Importantly, we observed the assumed interaction between evaluative information and task (Table 3; Fig. 5). Resolving this interaction, posthoc tests showed that a larger differentiation between negatively and neutrally associated faces was found for the evaluative compared with the perceptual task (*M*_difference_ = 0.38, *SD* = 0.97; *t*_(79)_ = 3.55, *p =* 0.001), and for the evaluative compared with the age task (*M*_difference_ = 0.47, *SD* = 1.23; *t*_(79)_ = 3.40, *p =* 0.001). No differences were found between the age and perceptual task (*M*_difference_ = −0.08, *SD* = 1.09; *t*_(79)_ = −0.69, *p =* 0.491). Regarding correlation analyses, we found no significant relationships between ERP amplitude differences and trait scores (Table 5; Fig. 5).

## Discussion

The present study investigated how negative evaluative information affects early (P1, N170), mid-latency (EPN), and late (LPP) ERP components depending on task conditions and individual differences in trait anxiety, neuroticism, and agreeableness. We found an effect of negative evaluative knowledge for the N170, independent of task condition, and interactions of evaluative information and task for the EPN and the LPP. Importantly, we found that low agreeableness increased ERP amplitudes for faces associated with negative evaluative information when faces were task-irrelevant.

We aimed to provide a systematic overview of how trait anxiety, neuroticism, or agreeableness modulate evaluative person knowledge ERP responses. Trait anxiety and neuroticism exhibit a high overlap (Bishop & Forster, 2013), both have been linked to an increased sensitivity to detect faces signaling threat (Chan et al., 2007; Doty et al., 2013; Andric et al., 2016; de Jong et al., 2009). Furthermore, for high trait anxiety, previous studies showed either increased early (for the P1, see Bar-Haim et al., 2005; Holmes et al., 2008; for the N170, see Williams et al., 2007) or attenuated EPN towards threat-related facial expressions (Holmes et al., 2008; Walentowska & Wronka, 2012). Moreover, for the EPN, we recently observed in a comparable sample that reduced fearful face processing for participants with high anxiety occurred specifically during perceptual distraction tasks (Steinweg et al., in press). In contrast to these studies, we found no systematic relationship between trait anxiety or neuroticism with ERP differences. It should be noted that emotional expressions, such as fearful faces, differ from neutral ones in specific spatial frequencies, which modulate early ERPs (Bruchmann et al., 2020) even in the absence of face information (Schindler et al., 2021). Thus, studies relating trait anxiety to increased ERPs for fearful expressions might show a higher early sensitivity to such emotion-specific frequencies, which are absent for the currently used neutral expressions. Given the reported increased processing of negative content during late processing stages in high neurotic participants (Gomez et al., 2002; Zhang et al., 2013; Ku et al., 2020; Zhang et al., 2015; but see Bartussek et al., 1996; Speed et al., 2015), we also do not exclude such effects per se for inherently neutral faces. Such relationships, however, might require a higher degree of emotional salience of the faces (i.e., either by more intense or extended learning experiences). Finally, while we can provide mostly moderate evidence against a relationship of trait anxiety and neuroticism and ERP differences, this evidence was only anecdotal for N170 effects during the perceptual discrimination task. In our attempt to provide an overview on possible relationships with individual traits and the necessity to correct for multiple comparisons, our sample size, while comparably large, might be not sensitive enough to detect small relationships.

In contrast, less agreeable participants showed a larger EPN amplitude for negative faces during perceptual distraction. Low agreeable individuals do not act prosocially when exposed to negative or aggression-related situations (Graziano et al., 2007; Meier et al., 2006). Following a different taxonomy, the callous-unemotional (CU) trait subfactor “aggression” correlates strongly with agreeableness (Poy et al., 2014). High CU traits exhibit a lack of guilt, shallow affect, and related to antisocial behavior. Participants with high CU scores showed reduced N170 and LPP responses to fearful expressions which have been related to low empathy and resulting impairment to detect fearful expressions (Brislin et al., 2018; Brislin & Patrick, 2019). Furthermore, violent offenders show a bias to detect anger in ambiguous anger-fearful expressions (Wegrzyn et al., 2017) which might explain a link between agreeableness and aggressive behavior (for a meta-analysis, see Jones et al., 2011). While these findings are only distantly related to our task, a preliminary explanation would be that agreeableness relates to a sensitivity to detect anger and hostility in faces, possibly showing antagonistic effects to trait anxiety. However, it is unclear why this sensitivity of low agreeable participants was limited to the perceptual task. Research on trait anxiety suggests stronger effects during implicit emotion processing tasks (see above), and this might be similar for agreeableness. This task-specificity might indicate that, although emotional information should not be attended to, low agreeable participants are distracted by such information and process emotional background information to some extent. Individual differences might play a negligible role when attention should be directed to the face or emotion. Alternatively, the pairing of negative evaluation and neutral faces might have been viewed as inconsistent information. For low agreeable participants, neutral faces paired with hostile information might have caused higher unexpectedness. Partly in line with this argument, physical salient distracters were recently found to increase the EPN in an object tracking task (Hoffman et al., 2020), and thus the EPN might share features with other attention-related N2 components, for example, the Mismatch Negativity. In summary, our study showed increased EPN differences between criminal and neutral faces for low agreeable participants when attention was directed away from the face information.

A secondary goal was to study the effects of attention tasks on ERP differences. Here, no effect of evaluative information was found for the P1, which was in line with our expectations and previous studies on affective context or evaluative background information for neutral faces (Wieser et al., 2014; Luo et al., 2016). The P1 is related to early stimulus detection and discrimination (Luck & Hillyard, 1994). While studies show very early modulations for neutral expressions for associated monetary gains (Hammerschmidt et al. 2017, but see Hammerschmidt et al. 2018) or classic conditioning (Rehbein et al., 2014), this finding indicates that evaluative information is not sufficient to increase P1 amplitudes. For the N170, an increased amplitude for negative evaluative information was found to be independent of the given task. Such effects have been reported previously when examining stereotypes or evaluative background information (Giménez-Fernández et al., 2020; Luo et al., 2016) and our subsample (Schindler et al., in press). For the EPN and LPP components, interactions between the attended feature and the evaluative information were found, showing significant effects only when attending to the evaluative information, partly explaining the mixed findings for these components in previous studies (Baum et al., 2018; Kissler & Strehlow, 2017; Luo et al., 2016; Suess et al., 2015). However, it has to be noted, in some studies EPN modulations are reported, while participants did not need to pay attention to the emotional information (Suess et al., 2015; Xu et al., 2016). We reason that presentation time might be important and the used short presentation time of the faces successfully avoided attention spillover to other face information (i.e., retrieving semantic information of the criminal/neutral background). A late differentiation between evaluative associations occurred pronounced when attention was directed to this evaluative information, which is in line with those studies with no significant evaluative LPP effects (Luo et al., 2016), and studies manipulating feature-based attention to threatening emotional expressions (Rellecke et al., 2012; Schindler et al., 2020), and is further in line with the hypothesized elaborative stimulus evaluation processes during this stage (Hajcak et al., 2009, 2010).

### Constraints on generality and future directions

We would like to note some limitations of the current study. While we examined an ecologically valid phenomenon, such evaluative information is often transmitted by rich media coverage, leading to prolonged exposure of individual faces and associated information across weeks or even months. Therefore, our findings might underestimate evaluative effects in everyday life. While we collected a comparably large sample, the sample size is still limited and thus we might have not sufficient sensitivity to detect smaller relationships. We used a rather short stimulus presentation, which possibly added to the clarity of our findings, and used specific tasks to manipulate attention to perceptual, facial, or emotional features. Future studies are needed, using other tasks to generalize our findings (e.g., attention to gender, face, or nationality information). Specifically, we used only male faces for background story purposes, while the sample was dominantly female. In this regard, future studies should aim to test sex differences but also resolve how depicted face-identity might interact with evaluative information (e.g., age, gender, ethnic background). Lastly, we only focused on the impact of threat-related biographical information, because it is reported to be highly effective (Suess et al., 2015; Schellhaas et al., 2020; Bublatzky et al., 2020a). However, ERP modulations might be not restricted to negative information, especially regarding individual differences in the relation between agreeableness and pro-social behavior. Thus, it seems promising to include positively associated faces in future studies, for which studies have shown modulations starting with the P1 component (Hammerschmidt et al., 2017).

## Conclusions

To the best of our knowledge, this is the first EEG study that systematically examined the effects of individual differences on the processing of faces associated with negative information. Most importantly, low agreeableness increased EPN responses to putative criminal faces under conditions of perceptual distraction, suggesting sensitivity to hostile faces. In contrast, we found no relationships of trait anxiety or neuroticism with ERP differences. Our finding validates a previous finding that negative evaluative knowledge increases N170 amplitudes task-independently, while for the EPN and LPP components effects are observed only for attention to the emotional information.

## Supplementary Information

ESM 1 (DOCX 815 kb)

## Footnotes

1 Due to scheduling of participants, eventually 82 usable EEG datasets were recorded, while analyses were registered for 80 datasets. We thus now excluded two datasets with partly incomplete behavioural datasets.

2 For young males we selected the following MPI FACES identities 025, 037, 049, 123, and for middle-aged males identities 058, 108, 178, and 179.
