# Supplementary material for: Electrophysiological responses to negative evaluative person-knowledge: Effects of individual differences
Source: Cogn Affect Behav Neurosci. 2021 Apr 13;21(4):822–36. doi: 10.3758/s13415-021-00894-w (PMC8354867; doi:10.3758/s13415-021-00894-w)
Supplement: Supplementary file 1 — (DOCX 815 kb) [file 13415_2021_894_MOESM1_ESM.docx]

**Electrophysiological responses to negative evaluative person-knowledge: Effects of individual differences**

**– Supplementary Materials –**

Claudia Krasowski^1*^, Sebastian Schindler^1,2*┼^, Maximilian Bruchmann^1,2^, Robert Moeck^1^, and Thomas Straube^1,2^

1. Institute of Medical Psychology and Systems Neuroscience, University of Muenster
2. Otto Creutzfeldt Center for Cognitive and Behavioral Neuroscience, University of Muenster

Corresponding author

*Corresponding author

^┼^Co-first authors

**Correspondence address**

Institute of Medical Psychology and Systems Neuroscience

University of Münster

Von-Esmarch-Str. 52, D-48149 Münster, Germany

e-mail: [sebastian.schindler@ukmuenster.de](mailto:sebastian.schindler@ukmuenster.de)

1. **Evaluative knowledge backstories**

For manipulating the associated evaluative knowledge to the neutral faces, we used two different background stories in the form of newspaper articles (see Supplementary Figure S1). After each article, four of the putative mentioned group members were closely portrayed and participants were instructed to remember these faces.

**English translation of the two backstories**

**Negative story**

*Colorado –* It is a horrible and brutal crime that can hardly be described to our readers. In Arriba, a small village two hours from Denver the dead bodies of two female girls were found. Her bodies were mutilated, handcuffed, and their throat cut. Before, their parents searched the whole night long to find the 12 and 13 years old aged two cousins. The two girls went for a walk with their dog. When they did not come back their relatives started with the search. In the early hours of the 8th of August, the parents found their horribly mutilated children. An absolute Horror! The Daily News reported that the two corps had various intersections, including their genitals. The agents are convinced that the two girls were raped and then mutilated and killed. On Sunday the police captured three suspected criminals (Mark P., Jackson T. und Tyler P.) who likely raped and murdered the two cousins. A fourth male (John T.), who is also accused of was involved in this horrible crime. He vanished and is currently searched by the police. Repeatedly, such cases of terrible violence and murder occur.

**Neutral story**

*Lauterbach –* Recently at the Lauterbach firefighter station, a teaching course for engineers of firefighter trucks started. The course was given by the fire inspector of the local city Jürgen Eifert and the training team included Jörg B (firefighter Lauterbach), Walter R (firefighter Alsfeld), and their assistants Martin O and Thomas C (firefighter Grebenhain). In total, the course took place on six days where a lot of different aspects were trained. In the theoretical part, a multitude of topics was discussed, including the objectives of the firefighter engineer, vehicle training, securing the load, legal issues, as well as special issues and right of passage, and the theoretical aspects of using the firefighter centrifugal pump and ventilation. The practical training included the use of different types of portable pumps and centrifugal pumps, which use was closely inspected. Further, the whole course was completed by a variety of different add-on themes, for example how to detect errors or malfunctions in the centrifugal pump and other machines. The course was finished by a practical examination which was done by the district fire chief Tony M. Eifert summarized that all members completed the course very well and the aim of the course was reached.

**
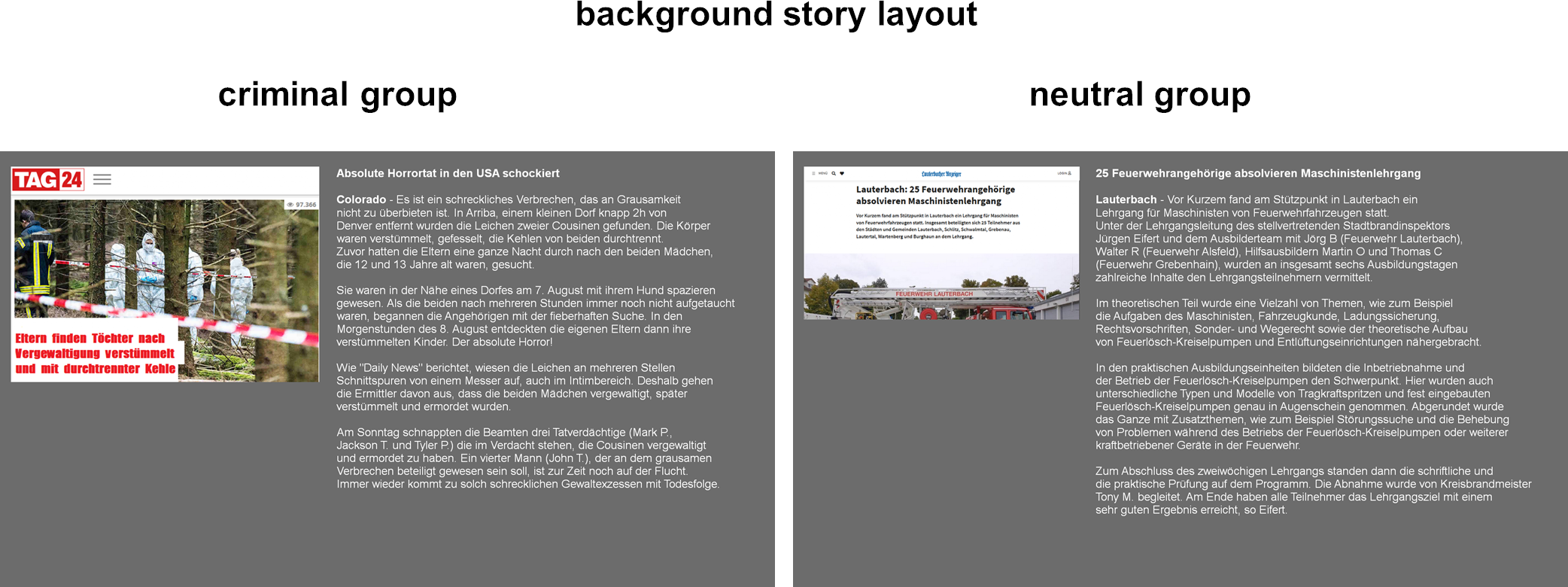
** **Supplementary Figure S1. Screenshots of the appearance of the two newspaper articles with negative or neutral content.**

**The German text of the two backstories**

**Negative story**

*Colorado -* Es ist ein schreckliches Verbrechen, das an Grausamkeit nicht zu überbieten ist. In Arriba, einem kleinen Dorf knapp zwei Stunden von Denver entfernt wurden die Leichen zweier Cousinen gefunden. Die Körper waren verstümmelt, gefesselt, die Kehlen von beiden durchtrennt. Zuvor hatten die Eltern eine ganze Nacht durch nach den beiden Mädchen, die 12 und 13 Jahre alt waren, gesucht. Sie waren in der Nähe eines Dorfes am 7. August mit ihrem Hund spazieren gewesen. Als die beiden nach mehreren Stunden immer noch nicht aufgetaucht waren, begannen die Angehörigen mit der fieberhaften Suche. In den Morgenstunden des 8. August entdeckten die eigenen Eltern dann ihre verstümmelten Kinder. Der absolute Horror! Wie die "Daily News" berichtet, wiesen die Leichen an mehreren Stellen Schnittspuren von einem Messer auf, auch im Intimbereich. Deshalb gehen die Ermittler davon aus, dass die beiden Mädchen vergewaltigt, später verstümmelt und ermordet wurden. Am Sonntag schnappten die Beamten drei Tatverdächtige (Mark P., Jackson T. und Tyler P.) die im Verdacht stehen, die Cousinen vergewaltigt und ermordet zu haben. Ein vierter Mann (John T.), der an dem grausamen Verbrechen beteiligt gewesen sein soll, ist zur Zeit noch auf der Flucht. Immer wieder kommt zu solch schrecklichen Gewaltexzessen mit Todesfolge.

**Neutral story**

*Lauterbach -* Vor kurzem fand am Stützpunkt in Lauterbach ein Lehrgang für Maschinisten von Feuerwehrfahrzeugen statt. Unter der Lehrgangsleitung des stellvertretenden Stadtbrandinspektors Jürgen Eifert und dem Ausbilderteam mit Jörg B (Feuerwehr Lauterbach), Walter R (Feuerwehr Alsfeld), Hilfsausbildern Martin O und Thomas C (Feuerwehr Grebenhain), wurden an insgesamt sechs Ausbildungstagen zahlreiche Inhalte den Lehrgangsteilnehmern vermittelt. Im theoretischen Teil wurde eine Vielzahl von Themen, wie zum Beispiel die Aufgaben des Maschinisten, Fahrzeugkunde, Ladungssicherung, Rechtsvorschriften, Sonder- und Wegerecht sowie der theoretische Aufbau von Feuerlösch-Kreiselpumpen und Entlüftungseinrichtungen nähergebracht. In den praktischen Ausbildungseinheiten bildeten die Inbetriebnahme und der Betrieb der Feuerlösch-Kreiselpumpen den Schwerpunkt. Hier wurden auch unterschiedliche Typen und Modelle von Tragkraftspritzen und fest eingebauten Feuerlösch-Kreiselpumpen genau in Augenschein genommen. Abgerundet wurde das Ganze mit Zusatzthemen, wie zum Beispiel Störungssuche und die Behebung von Problemen während des Betriebs der Feuerlösch-Kreiselpumpen oder weiterer kraftbetriebener Geräte in der Feuerwehr. Zum Abschluss des zweiwöchigen Lehrgangs standen dann die schriftliche und die praktische Prüfung auf dem Programm. Die Abnahme wurde von Kreisbrandmeister Tony M. begleitet. Am Ende haben alle Teilnehmer das Lehrgangsziel mit einem sehr guten Ergebnis erreicht, so Eifert.

1. **Results from exploratory linear regressions models including all examined questionnaires**

We explored possible influences of all eight questionnaire measures on ERP differences, including the BDI-II score, the STAI state and trait score, and the five NEO-FFI trait scores (Hautzinger et al., 2009; Körner et al., 2008; Spielberger et al., 1999). Here, all models did not show a significant adjusted R² (see Supplementary Tables S1-S4), positive relationships were seen for the P1 with trait anxiety (age task), for the N170 for extraversion (perceptual task), for the EPN with agreeableness (perceptual task) and the LPP with neuroticism (emotion task).

**Supplementary Table S1: Linear regression models on P1 differences including all examined questionnaires**

|  | P1 difference perceptual task | | P1 difference age task | | P1 difference emotion task | |
| --- | --- | --- | --- | --- | --- | --- |
| Model | R²  (RMSE) | Adjusted R²  (*p-*value) | R²  (RMSE) | Adjusted R²  (*p-*value) | R²  (RMSE) | Adjusted R²  (*p-*value) |
|  | .118  (0.743) | .018  (.322) | .102  (0.677) | .001  (.435) | .098  (0.694) | -.004  (.473) |
| Coefficient | standartized β  (Std error) | *t*-value  (*p*-Value) | standartized β  (Std error) | *t*-value  (*p*-Value) | standartized β  (Std error) | *t*-value  (*p*-Value) |
| Intercept^a^ | -0.636  (1.104) | -0.577  (.566) | -1.013  (1.007) | -1.007  (.318) | -0.139  (1.032) | -0.135  (.893) |
| BDI-II | 0.052  (0.023) | 0.327  (.744) | -0.168  (0.021) | -1.042  (.301) | -0.021  (0.022) | -0.133  (.895) |
| STAI state | 0.085  (0.014) | 0.670  (.505) | -0.060  (0.012) | -0.470  (.640) | 0.102  (0.013) | 0.789  (.433) |
| STAI trait | -0.084  (0.022) | -0.371  (.712) | **0.571**  **(0.020)** | **2.500**  **(.015)** | 0.257  (0.021) | 1.124  (.265) |
| neuroticism | 0.054  (0.209) | 0.259  (.796) | -0.376  (0.191) | -1.793  (.077) | -0.259  (0.195) | -1.234  (.221) |
| extraversion | 0.224  (0.154) | 1.838  (.070) | 0.096  (0.140) | 0.779  (.438) | 0.131  (0.144) | 1.064  (.291) |
| openness | 0.021  (0.119) | 0.172  (.864) | -0.158  (0.109) | -1.270  (.208) | -0.130  (0.111) | -1.044  (.300) |
| agreeableness | 0.151  (0.169) | 1.167  (.247) | -0.079  (0.154) | -0.602  (.549) | -0.209  (0.158) | -1.594  (.115) |
| conscientiousness | -0.198  (0.165) | -1.441  (.154) | 0.072  (0.151) | 0.521  (.604) | -0.042  (0.154) | -0.299  (.766) |

Note. ^a^ = unstandardized beta for the intercept; RMSE = standard error of the estimate.

**Supplementary Table S2: Linear regression models on N170 differences including all examined questionnaires**

|  | N170 difference perceptual task | | N170 difference age task | | N170 difference emotion task | |
| --- | --- | --- | --- | --- | --- | --- |
| Model | R²  (RMSE) | Adjusted R²  (*p-*value) | R²  (RMSE) | Adjusted R²  (*p-*value) | R²  (RMSE) | Adjusted R²  (*p-*value) |
|  | .172  (0.643) | .079  (.082) | .063  (0.593) | -.043  (.777) | .052  (0.666) | -.055  (.865) |
| Coefficient | standartized β  (Std error) | *t*-value  (*p*-Value) | standartized β  (Std error) | *t*-value  (*p*-Value) | standartized β  (Std error) | *t*-value  (*p*-Value) |
| Intercept^a^ | -0.826  (0.956) | -0.864  (.390) | -0.597  (0.882) | -0.677  (.501) | -1.269  (0.989) | -1.283  (.204) |
| BDI-II | -0.139  (0.020) | -0.895  (.374) | -0.238  (0.018) | -1.441  (.154) | -0.088  (0.021) | -0.528  (.599) |
| STAI state | 0.069  (0.012) | 0.560  (.577) | 0.098  (0.011) | 0.743  (.460) | 0.052  (0.012) | 0.392  (.696) |
| STAI trait | 0.006  (0.019) | 0.029  (.977) | 0.327  (0.018) | 1.402  (.165) | 0.114  (0.020) | 0.487  (.628) |
| neuroticism | -0.047  (0.181) | -0.232  (.817) | -0.246  (0.167) | -1.147  (.255) | 0.087  (0.187) | 0.403  (.688) |
| extraversion | **0.255**  **(0.133)** | **2.158**  **(.034)** | -0.008  (0.123) | -0.063  (.950) | -0.074  (0.138) | -0.583  (.562) |
| openness | -0.220  (0.103) | -1.839  (.070) | -0.088  (0.095) | -0.688  (.494) | -0.018  (0.107) | -0.137  (.891) |
| agreeableness | 0.193  (0.146) | 1.539  (.128) | 0.010  (0.135) | 0.076  (.939) | 0.084  (0.151) | 0.624  (.534) |
| conscientiousness | -0.107  (0.143) | -0.804  (.424) | -0.070  (0.132) | -0.490  (.625) | 0.128  (0.148) | 0.893  (.375) |

Note. ^a^ = unstandardized beta for the intercept; RMSE = standard error of the estimate.

**Supplementary Table S3: Linear regression models on EPN differences including all examined questionnaires**

|  | EPN difference perceptual task | | EPN difference age task | | EPN difference emotion task | |
| --- | --- | --- | --- | --- | --- | --- |
| Model | R²  (RMSE) | Adjusted R²  (*p-*value) | R²  (RMSE) | Adjusted R²  (*p-*value) | R²  (RMSE) | Adjusted R²  (*p-*value) |
|  | .170  (0.666) | .077  (.087) | .090  (0.757) | -.012  (.538) | .058  (0.803) | -.048  (.817) |
| Coefficient | standartized β  (Std error) | *t*-value  (*p*-Value) | standartized β  (Std error) | *t*-value  (*p*-Value) | standartized β  (Std error) | *t*-value  (*p*-Value) |
| Intercept^a^ | -2.332  (0.990) | -2.355  (.021) | -1.482  (1.125) | -1.317  (.192) | -1.826  (1.194) | -1.529  (.131) |
| BDI-II | 0.159  (0.021) | 1.025  (.309) | -0.002  (0.024) | -0.015  (.988) | 0.200  (0.025) | 1.209  (.231) |
| STAI state | -0.027  (0.012) | -0.220  (.826) | 0.192  (0.014) | 1.483  (.143) | 0.106  (0.015) | 0.808  (.424) |
| STAI trait | 0.100  (0.020) | 0.457  (.649) | 0.163  (0.022) | 0.708  (.481) | 0.029  (0.024) | 0.124  (.901) |
| neuroticism | -0.154  (0.188) | -0.764  (.447) | -0.334  (0.213) | -1.582  (.118) | -0.076  (0.226) | -0.352  (.726) |
| extraversion | 0.106  (0.138) | 0.893  (.375) | 0.128  (0.157) | 1.033  (.305) | -0.062  (0.166) | -0.495  (.622) |
| openness | 0.041  (0.107) | 0.342  (.734) | 0.015  (0.122) | 0.117  (.907) | 0.084  (0.129) | 0.655  (.514) |
| agreeableness | **0.351**  **(0.151)** | **2.797**  **(.007)** | -0.036  (0.172) | -0.272  (.786) | -0.004  (0.183) | -0.032  (.974) |
| conscientiousness | 0.116  (0.148) | 0.868  (.388) | 0.072  (0.168) | 0.514  (.609) | 0.245  (0.179) | 1.723  (.089) |

Note. ^a^ = unstandardized beta for the intercept; RMSE = standard error of the estimate.

**Supplementary Table S4: Linear regression models on LPP differences including all examined questionnaires**

|  | LPP difference perceptual task | | LPP difference age task | | LPP difference emotion task | | |
| --- | --- | --- | --- | --- | --- | --- | --- |
| Model | R²  (RMSE) | Adjusted R²  (*p-*value) | R²  (RMSE) | Adjusted R²  (*p-*value) | R²  (RMSE) | | Adjusted R²  (*p-*value) |
|  | .049  (0.670) | -.058  (.881) | .049  (0.916) | -.058  (.881) | .146  (0.864) | | .049  (.168) |
| Coefficient | standartized β  (Std error) | *t*-value  (*p*-Value) | standartized β  (Std error) | *t*-value  (*p*-Value) | standartized β  (Std error) | *t*-value  (*p*-Value) | |
| Intercept^a^ | 1.486  (0.996) | 1.492  (.140) | 0.627  (1.361) | 0.461  (.646) | 4.158  (1.284) | | **3.238**  **(.002)** |
| BDI-II | -0.004  (0.021) | -0.026  (.979) | 0.154  (0.028) | 0.923  (.359) | -0.129  (0.027) | | -0.821  (.414) |
| STAI state | -0.067  (0.012) | -0.507  (.614) | -0.015  (0.017) | -0.110  (.913) | -0.230  (0.016) | | -1.837  (.070) |
| STAI trait | -0.334  (0.020) | 1.421  (.160) | -0.068  (0.027) | -0.289  (.774) | -0.423  (0.026) | | -1.896  (.062) |
| neuroticism | 0.314  (0.189) | 1.456  (.150) | -0.067  (0.258) | -0.311  (.757) | **0.507**  **(0.243)** | | **2.479**  **(.016)** |
| extraversion | -0.077  (0.139) | -0.606  (.547 | -0.187  (0.190) | -1.479  (.144) | -0.079  (0.179) | | -0.655  (.514) |
| openness | 0.067  (0.108) | 0.520  (.604) | 0.026  (0.147) | 0.205  (.838) | 0.034  (0.139) | | 0.277  (.783) |
| agreeableness | 0.021  (0.152) | 0.155  (.877) | 0.133  (0.208) | 0.985  (.328) | -0.085  (0.196) | | -0.665  (.508) |
| conscientiousness | -0.114  (0.149) | -0.799  (.427) | -0.046  (0.204) | -0.322  (.749) | -0.181  (0.192) | | -1.336  (.186) |

Note. ^a^ = unstandardized beta for the intercept; RMSE = standard error of the estimate.
